# Editorial: Rehabilitation strategies for musculoskeletal disorders

**DOI:** 10.3389/fresc.2026.1942211

**Published:** 2026-08-07

**Authors:** Angelo Alito, Simona Portaro

**Affiliations:** 1 Brain Mapping Lab, Department of Biomedical, Dental Sciences and Morphological and Functional Imaging, University of Messina, Messina, Italy; 2 Department of Physical and Rehabilitation Medicine, University Hospital “G. Martino”, Messina, Italy

**Keywords:** exercise therapy, functional recovery, musculoskeletal rehabilitation, passive modalities, rehabilitation, telerehabilitation

Musculoskeletal disorders are a leading cause of global disability, placing a significant physical, emotional and financial strain on individuals and healthcare systems. For decades, the rehabilitative landscape has been shaped by a persistent dichotomy between passive treatments delivered to patients and active exercises performed by them. However, the contemporary paradigm of physical and rehabilitation medicine is moving beyond this opposition. Clinical excellence is not achieved by choosing one modality over another, but by mastering their sequential temporal integration.

This Research Topic brings together clinical trials, systematic reviews, case studies and protocols that collectively demonstrate a unifying principle: passive interventions and advanced biophysical technologies facilitate biological and neurological processes. By reducing acute nociceptive drive and restoring tissue compliance, they create an opportunity for patients to safely and early transition into progressive active exercise, thereby optimizing adherence and long-term functional recovery. The contributions can be read as three consecutive phases of a single continuum, as summarized in Figure 1.

**Figure 1:**
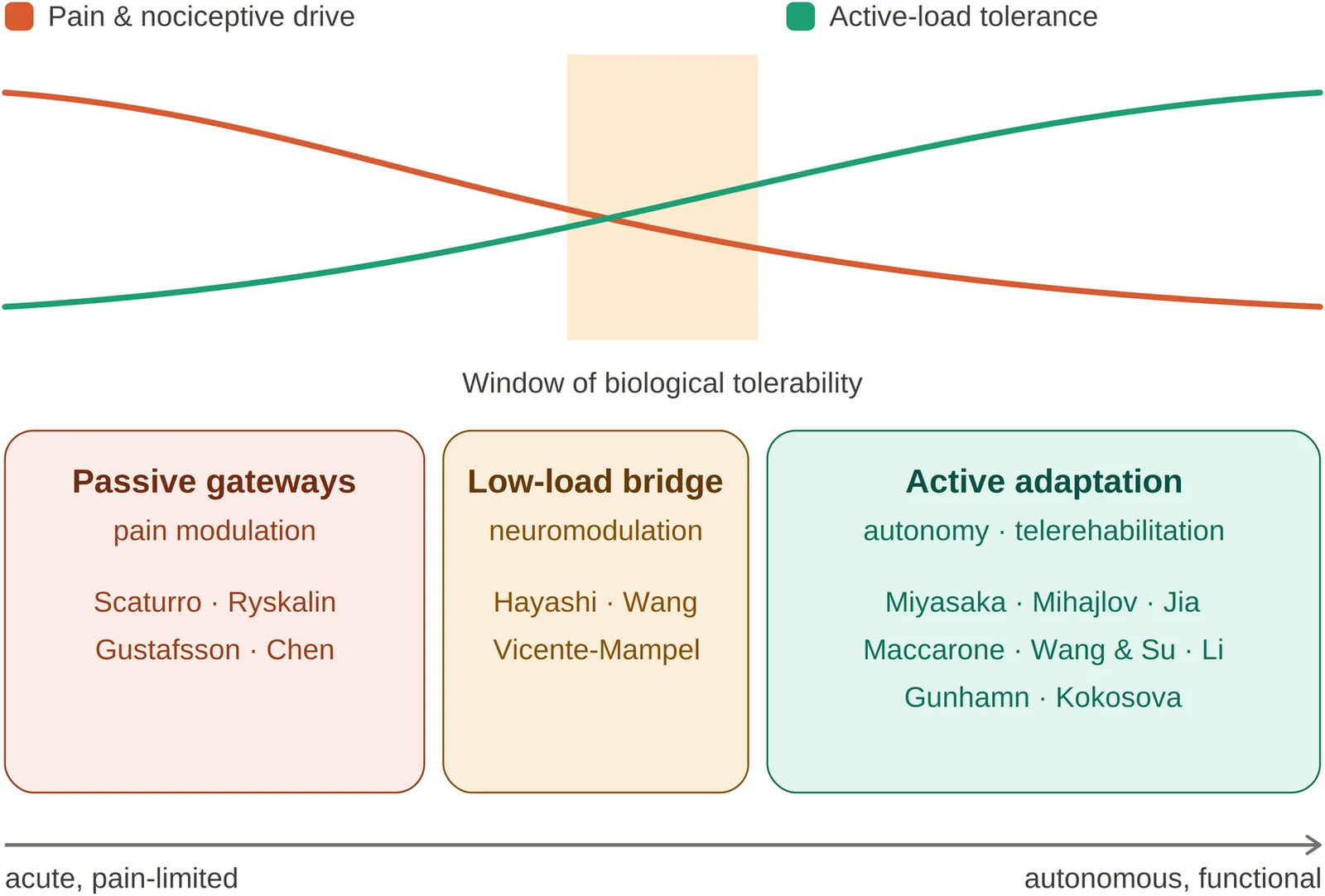
The rehabilitation continuum. Passive and technological modalities reduce nociceptive drive and arthrogenic inhibition, creating a period during which progressively active, patient-led loading becomes possible. The fifteen contributing studies are distributed across the three sequential phases.

## Phase 1 — tissue release and pain modulation: passive and technological gateways

The initial management of acute or recalcitrant musculoskeletal pathology is often obstructed by pain, mechanical restriction, and arthrogenic inhibition, which prevent active movement. Precise passive or technological modalities can prepare the local biological substrate. A focused-energy thread runs through three of the contributions. In lateral epicondylitis, Scaturro et al. show that combining focal extracorporeal shock wave therapy (ESWT) with therapeutic exercise and an oral nutraceutical (hyaluronic acid, collagen, vitamin C, and manganese) produces the greatest short-term gains, reducing pain and common extensor tendon thickness while improving grip strength by 30 days. Ryskalin et al. extend ESWT to medial epicondylitis complicated by ulnar nerve instability, reporting durable pain relief beyond two years with no neural adverse effects, while Gustafsson and Ryman Augustsson document patient-adapted focused shock waves in thumb carpometacarpal osteoarthritis, with QuickDASH falling from 20.5 to 2.3 and grip strength rising by nearly 30% at one year. Complementing these energy-based approaches, the meta-analysis by Chen et al. finds that adding acupuncture to rehabilitation after arthroscopic rotator cuff repair lowers pain and improves Constant–Murley scores and range of motion, although the pooled evidence is of low certainty. Across upper-limb and shoulder targets, the shared mechanism is the same: a controlled interval of analgesia and mechanotransductive stimulation that renders subsequent active loading tolerable.

## Phase 2 — bridging the gap: neuromodulation and controlled low-load interventions

Once peripheral nociception is attenuated, the nervous system frequently remains in a state of altered excitability and protective motor inhibition. Hybrid strategies can overcome this central resistance without overloading healing tissue. At the peripheral end, Hayashi et al. illustrate how ultrasound-guided neural mobilization added to conventional therapy restored the longitudinal gliding velocity of the posterior interosseous nerve and achieved complete motor recovery by day 109 after ganglion cyst aspiration, mechanically unlocking the nerve before active re-education. Centrally, the randomized protocol by Vicente-Mampel et al. applies anodal transcranial direct current stimulation over the primary motor cortex during early rehabilitation after anterior cruciate ligament reconstruction (ACLR), aiming to modulate corticospinal drive and counter arthrogenic muscle inhibition; outcomes are awaited. Between neuromodulation and conventional loading, the best-evidence summary by Wang et al. consolidates practical recommendations for blood flow restriction training after ACLR — spanning applicable population, contraindications, training intensity and frequency, monitoring, and safety — so that low-load exercise with an individualized limb occlusion pressure can be applied as a controlled stimulus when high mechanical loads remain contraindicated.

## Phase 3 — sustainability, autonomy, and active functional adaptation

The final phase shifts decisively toward active empowerment; early gains endure only through the functional adaptations that follow. Two studies anchor active capacity to patient-reported quality of life: Miyasaka et al. identify weight-bearing ankle dorsiflexion during a deep squat, together with gait speed, as independent predictors of quality of life after ankle fracture surgery, and Mihajlov et al. show that recovery of hip extension strength at twelve weeks is the sole significant predictor of quality of life after total hip arthroplasty. Sustaining such gains increasingly depends on decentralized delivery. The meta-analysis by Jia et al. confirms that tele-rehabilitation improves physical function in knee osteoarthritis, even where its effect on pain does not reach significance; Maccarone et al. reveal a temporal nuance in Long Covid balance rehabilitation, with supervised home-based exercise producing faster initial improvement and thermal aquatic rehabilitation yielding more gradual, durable postural control; and Wang and Su demonstrate that a fully online, home-based Schroth–Pilates program reduced the Cobb angle by 5.39° with high adherence in adolescents with idiopathic scoliosis, most markedly in younger patients. The durability of active care also rests on systemic and methodological readiness. Gunhamn et al. show, in patellofemoral pain, that exercise intensity and range of motion are inadequately reported in most primary studies, threatening reproducibility and adherence, while Li et al. map the strong evidence-based-practice readiness of orthopedic nurses as an organizational prerequisite for delivering complex exercise programs. Finally, Kokosova et al. close the loop in chronic non-specific low back pain: an eighteen-week hybrid home program improved strength, endurance, and disability without altering paraspinal muscle morphology on MRI, confirming that recovery is driven primarily by neuromuscular reactivation rather than structural change.

## A unified chronological framework

Taken together, these contributions demonstrate that optimal functional recovery is not exclusively passive or active, but rather a finely tuned chronological sequence. As Topic Editors, we advocate a unified model in which passive modalities and biophysical technologies are strategically deployed to minimise early tissue stress and pain. This creates a stable foundation on which to build patient-led, precise and digitally supported active training. In our view, this closed-loop approach is the most reliable way to guarantee long-term adherence, clinical efficacy, and sustainable musculoskeletal health.

